# A Polymer/Oil Based Nanovaccine as a Single-Dose Immunization Approach

**DOI:** 10.1371/journal.pone.0062500

**Published:** 2013-04-22

**Authors:** Sara Vicente, Belen Diaz-Freitas, Mercedes Peleteiro, Alejandro Sanchez, David W. Pascual, Africa Gonzalez-Fernandez, Maria J. Alonso

**Affiliations:** 1 Pharmacy and Pharmaceutical Technology Department, Center for Research in Molecular Medicine and Chronic Diseases (CIMUS), University of Santiago de Compostela, Santiago de Compostela, Spain; 2 Immunology, Institute of Biomedical Research (IBIV), Biomedical Research Center (CINBIO), University of Vigo, Vigo, Spain; 3 Pharmacy and Pharmaceutical Technology Department, School of Pharmacy, University of Santiago de Compostela, Santiago de Compostela, Spain; 4 Health Research Institute of Santiago de Compostela (IDIS), Santiago de Compostela, Spain; 5 Department of Immunology and Infectious Diseases, Montana State University, Bozeman, Montana, United States of America; 6 Department of Infectious Diseases & Pathology, University of Florida, Gainesville, Florida, United States of America; The Ohio State University, United States of America

## Abstract

The recognized necessity for new antigen delivery carriers with the capacity to boost, modulate and prolong neutralizing immune responses prompted our approach, in which we describe a multifunctional nanocarrier consisting of an oily nanocontainer protected by a polymeric shell made of chitosan (CS), named CS nanocapsules (CSNC). The CS shell can associate the antigen on its surface, whereas the oily core might provide additional immunostimulating properties. In this first characterization of the system, we intended to study the influence of different antigen organizations on the nanocarrier's surface (using the recombinant hepatitis B surface antigen –rHBsAg– as a model antigen) on their long-term immunopotentiating effect, without any additional immunostimulant. Thus, two prototypes of antigen-loaded CSNC (CSNC+ and CSNC−), exhibiting similar particle size (200 nm) and high antigen association efficiency (>80%), were developed with different surface composition (polymer/antigen ratios) and surface charge (positive/negative, respectively). The biological evaluation of these nanovaccines evidenced the superiority of the CSNC+ as compared to CSNC- and alum-rHBsAg in terms of neutralizing antibody responses, following intramuscular vaccination. Moreover, a single dose of CSNC+ led to similar IgG levels to the positive control. The IgG1/IgG2a ratio suggested a mixed Th1/Th2 response elicited by CSNC+, in contrast to the typical Th2-biased response of alum. Finally, CSNC+ could be freeze-dried without altering its physicochemical properties and adjuvant effect *in vivo*. In conclusion, the evaluation of CSNC+ confirms its interesting features for enhancing, prolonging and modulating the type of immune response against subunit antigens, such as rHBsAg.

## Introduction

Aluminum salts (alum), mainly in the form of aluminum hydroxide (Al(OH)_3_) or aluminum phosphate (AlPO_4_), have been traditionally used worldwide (since 1930s) as adjuvants in many vaccines [1]. Unfortunately, despite their wide use, these adjuvants exhibit important drawbacks, such as inflammatory reactions at the injection site, irreversible loss of potency upon freezing and induction of strong biased Th2-type immune responses [2]. Consequently, many efforts have been oriented to the search of new adjuvants and delivery carriers, which could help induce, strengthen, and simultaneously prolong the immune response. Ideally, the delivery carrier could also contribute to the development of single-dose vaccines capable of generating protective immunity upon only one antigenic exposure. This is especially relevant for subunit antigens, characterized by their improved safety profile, but low immunogenicity, which usually require multiple doses to assure immunological protection [3].

A promising strategy towards this goal relies in the design of polymeric nanocarriers, which are known to protect the associated antigen from degradation, facilitate antigen uptake by antigen-presenting cells (APCs), and control antigen release [4]. Within this frame, the increasing understanding of the influence of the nanocarrier characteristics (composition, size, charge) on their effectiveness is gradually paving the way to the rational design of nanovaccines [5]. For instance, both size and surface properties of polymeric nanocarriers are known to have a critical influence on the uptake and activation of APCs [6] and, ultimately, on the type of immune response generated (preferentially cellular vs. humoral) [7], [8].

Chitosan (CS) is one of the most commonly used biomaterials in vaccine delivery. So far, its use has been found particularly promising for mucosal immunization because of its mucoadhesive and penetration enhancing properties [9]. In fact, chitosan itself, as a dry powder mixed with monophosphoryl lipid A (MPLA) and the antigen, is currently ongoing phase I of clinical evaluation for intranasal vaccination against norovirus [10], [11]. On the other hand, our group, among others, has developed CS nanoparticles specifically adapted for the protection and delivery of antigens [12]. In particular, CS nanoparticles have demonstrated great potential for nasal vaccination [13]–[17]. Recently, their application as vaccine adjuvants has also been investigated against a variety of protein and DNA-encoded antigens [14], [18]–[21], suggesting additional immunological properties of these CS-based nanocarriers.

Taking this background information into account, the aim of our work has been to design a new CS-based nanocarrier that might offer advanced properties in terms of antigen localization and the possibility to incorporate additional immunomodulating agents. This new nanocarrier, named CS nanocapsules (CSNC), has a core-corona architecture, which enables the surface presentation of different types of antigens while co-delivering immunoactive molecules included in the oily core. However, in the present work, we intended to study the effect of the organization of the antigen molecules on the nanocarrier's surface, without including any additional immunostimulant, on their ability to promote antigen specific immune responses. For this purpose, we have selected the recombinant hepatitis B surface antigen (rHBsAg, since now HB in the text) as a model antigen, which could benefit from this technology. Therefore, in our aim to explore the potential of CSNC as adjuvant and single-dose vaccine formulation, we have developed and evaluated different HB-surface assembled CSNC exhibiting different surface characteristics (both surface charge and composition) Overall, we have designed a formulation able to elicit long-lasting and protective immune responses against subunit antigens, such as the HB, in order to offer an alternative to alum as adjuvant agent, as well as possibly reduce the number of doses to elicit long-lasting immune protection.

## Materials and Methods

### Ethics statement

Immunization studies involving fresh formulations of CSNC prototypes were conducted in the University of Vigo (Spain), and all protocols were adapted to the guidelines of the Spanish regulations (Royal Decree 1201/2005) regarding the use of animals in scientific research and under approval of the ethical committee of the University of Vigo. In the case of freeze-dried CSNC+ (trehalose 5%), the dried formulation was properly arranged and shipped to Montana State University (Bozeman, MT) where the biological evaluation was performed. All animal care and procedures were in accordance with institutional policies for animal health and well-being and approved by Montana State University Institutional Animal Care and Use Committee (IACUC) under protocol 58.

Female BALB/c mice (4–5 weeks old) were housed in filter-top cages in a 12 h light/12 h dark cycle with constant temperature environment of 22°C and provided with food and water *ad libitum*.

### Materials

Ultrapure chitosan (CS) hydrochloride salt (Protasan UP CL 113, MW 125 kDa, acetylation degree of 14%) was purchased from Novamatrix (Sandvika, Norway). Miglyol® 812 (M812) is a neutral oil composed of triglycerides of medium chain fatty acids (6–8 C) and was donated by Sasol Germany GmbH (Witten, Germany). The emulsifier soybean L-α-lecithin Epikuron 145V was a gift from Cargill (Barcelona, Spain). The recombinant hepatitis B surface antigen (rHBsAg or HB) (MW 24 kDa) was kindly donated by Shantha Biotechnics Ltd. (Hyderabad, India) as an aqueous suspension in PBS containing a protein concentration of 0.16 mg/ml.

### Preparation of HB-surface-assembled chitosan nanocapsules and physicochemical characterization

Blank chitosan nanocapsules (CSNC) were prepared by the solvent displacement technique, as previously reported ([22], **Protocol S1**). The formation of HB-surface-assembled CSNC was achieved by the strong electrostatic interaction between the cationic polysaccharidic surface of CSNC and the negatively charged particle antigen (−20 mV in water). For this purpose, HB stock solution was desalted and concentrated to 0.5 mg/mL by ultrafiltration (Amicon Ultra4, Millipore; Cork, Ireland). The resulting HB aqueous solution was immediately mixed with blank CSNC (CS concentration 1 mg/mL) and incubated for 1 hour at room temperature. Several nanosystems were prepared following two different strategies (**Figure S1**): 1) increasing the antigen amount (25, 50, 100, 250 µg) in the formulation or 2) maintaining constant the antigen amount (25 µg), but changing the CSNC concentration. Following these two methods, it was possible to prepare a series of nanosystems defined by their CSNC:HB ratio, ranging from 1∶0.025 to 1∶12.8.

The particle size and polydispersity index were measured by photon correlation spectroscopy (PCS) and ζ potential by laser doppler anemometry (LDA).

### Quantification of HB association to CSNC

The amount of HB associated to CSNC surface was indirectly quantified by measuring the concentration of free antigen remaining in supernatant after ultracentrifugation (42000× g, 1 h, 15°C) of the nanostructures. An ELISA commercial kit (Murex HBsAg Version 3, Murex Biotech Ltd; Dartford, UK), was used to quantify HB concentration in the samples. The analysis protocol was conducted as specified by the manufacturer. The association efficiency for HB (A.E. %) was then calculated by the difference between the concentration of free antigen detected in the supernatant and the total concentration in the initial suspension.

### Stability of cationic HB-surface-assembled CSNC

#### Storage at 4°C of CSNC+ aqueous suspension

The stability of the CSNC+ suspension was assessed during storage at 4°C. Samples were collected each week during one month. Particle size and HB association to CSNC were analyzed according to the techniques already described.

#### Freeze-drying of CSNC+

The freeze-drying technique, or lyophilization, was used to enhance stability of CSNC+ by converting the aqueous suspension into a dry powder. For this purpose, prototype CSNC+ was freeze-dried in the presence of different sugar cryoprotectants (sucrose and trehalose) at concentrations 0, 1, 2.5, and 5%. CSNC+ suspension and cryoprotectant solution were mixed 1∶1 (v:v) in 5 ml freeze-drying glass vials. Vials were slowly frozen at −20°C and then placed on the stainless-steel shelf plates of the Labconco Freeze Dry System (Kansas City, MI), operating at −35°C. The primary drying (sublimation) was carried out at this temperature under high vacuum (10^−3^ mBar) for approximately 40 hours. The second drying step (moisture desorption) lasted 8 hours, during which the temperature gradually rose until +20°C under vacuum. The final freeze-dried product was reconstituted with ultrapure water by gentle pipette mixing and analyzed for its physicochemical properties.

### Vaccination studies

#### HB-alum preparation

HB adsorbed to aluminum hydroxide (HB-alum) was used as positive control. HB and aluminum hydroxide (Alhydrogel^TM^; Sigma-Aldrich, St. Louis, MO) solutions were incubated in a volumetric ratio 3∶1 (HB:alum) for 30 minutes at 4°C under moderate agitation. Then, the suspension was centrifuged (10000× g, 10 minutes, 4°C), and the pellet was resuspended in adequate volume of isotonic saline solution.

#### Immunizations and sample collection

The adjuvant capacity of CSNC was evaluated either in their fresh or freeze-dried forms. Groups of 10 female BALB/c mice were immunized with 10 μg of HB incorporated in the selected fresh CSNC:HB prototypes (CSNC− and CSNC+) or adsorbed to alum, at weeks 0 and 4 (two doses) or in a single dose. Formulations of CSNC and HB-alum were injected intramuscularly on the posterior leg of mice while animals were conscious. Subsequent sampling was performed monthly, post-primary immunization, during a total period of 27 weeks. Blood samples were collected from the maxillary vein without anesthesia.

For the biological evaluation of freeze-dried formulation, mice were randomly distributed in two groups of 10 animals and then immunized intramuscularly with reconstituted freeze-dried formulation or HB-alum as control following a boost-dose schedule (0 and 4 weeks). For reconstitution of freeze-dried formulations, 0.5 mL of ultrapure water was added, and gentle pipette mixing was applied in order to properly disperse the nanocapsules. Blood samples were collected at selected time points post-immunization (days 28, 56, 84, 112) and specific serum anti-HB IgG titers were analyzed by ELISA.

#### Measurement of specific immunoglobulin responses

Serum anti-HB IgG endpoint titers were measured by ELISA. Maxisorp microtiter wells were coated with 5 µg/mL of HB in carbonate buffer (pH 9.6) overnight at 4°C. Plates were then blocked with BSA 1% in PBS for 1 hour at 37°C in order to minimize non-specific interactions. Serum samples and a mouse IgG monoclonal antibody directed against HB (Biokit; Barcelona, Spain) (used as control in the calibration curve) were serially diluted and incubated for 2 hours at 37°C. All serum samples were tested at least twice and in duplicate. Control rabbit antiserum of known concentration (mIU/mL) (Acris Antibodies GmbH; Hiddenhausen, Germany) was used in order to transform serum titers into international units (IU). Goat anti-mouse and anti-rabbit IgG conjugated with horseradish peroxidase (Southern Biotech; Birmingham, AL) were added to each well and incubated for 1 hour at 37°C. Bound antibodies were revealed with ABTS and the titers were expressed in µg/ml or in mIU/mL.

Antigen specific IgG subclasses (G1 and G2a) were also quantified in mouse serum in order to know the IgG1/IgG2a ratio. For this study, pooled sera from all mice from each group were prepared and analyzed following a similar ELISA protocol described for total anti-HB specific IgG. In this case, polyclonal goat anti-mouse IgG1 and IgG2a antibodies, both conjugated with horseradish peroxidase (Southern Biotech; Birmingham, AL), were used as secondary antibodies. Then, a ratio between IgG1/IgG2a was calculated in relation to the optical density levels.

#### Statistical analysis

The analysis of variance (ANOVA) was performed using Statgraphics Plus 5.1. Tukey post-hoc analysis was employed to establish significant differences between groups. Differences were considered significant at a level of p<0.05.

## Results

### Development and characterization of HB-surface-assembled chitosan nanocapsules

**Figure 1:**
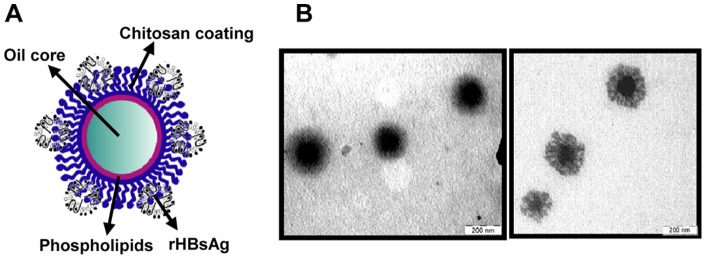
Morphology of CSNC. (A) Structure of HB-surface assembled CSNC showing the different components of the system. (B) TEM micrographs of blank CSNC (left) and HB-surface assembled CSNC (right).

For the purpose of loading HB onto CS nanocapsules (CSNC), we first prepared blank nanocapsules, and then the antigen was associated to their polymeric surface. Blank CSNC composed of an oily core of Miglyol^®^ 812 (M812) and lecithin and a CS shell were obtained by the solvent displacement technique. The resulting CSNC exhibited a particle size in the nanometer range (around 200 nm) with spherical shape and high positive ξ potential provided by the CS coating (**Table 1** and **Figure 1**).

**Table 1:** Physicochemical characterization of positively charged CSNC:HB prototypes.

CSNC:HB ratio	HB (μg)	Size (nm)	PdI	ζ potential (mV)	A.E. (%)
**Blank**	-	196±12	<0.2	+45±1	-
**1:0.025**	25	253±11	<0.2	n.d.	60±3
**1:0.05**	50	254±17	<0.2	n.d.	55±2
**1:0.1**	100	226±5	<0.2	n.d.	63±2
**1:0.25**	**250**	**245±53**	**<0.2**	**+41±7**	**78±3**

Results are presented as mean ± SD. *CSNC: chitosan nanocapsules; HB: recombinant hepatitis B surface antigen; PdI: polydispersity index; A.R.: association efficiency; n.d.: not determined*.

Because of the cationic nature of the polymeric coating, it was possible to associate negatively charged particulated viral proteins, such as the HB (22 nm size and ζ potential of −20 mV), to the surface of the CSNC. In order to identify the most adequate association conditions, we prepared different prototypes displaying different surface properties by associating the antigen at different ratios CSNC:HB.

Incubating increasing amounts of HB (25, 50, 100, 250 μg) in a 1 mL suspension of blank CSNC (CS concentration of 1 mg/mL) resulted in a series of nanosystems with CSNC:HB ratios between 1∶0.025 and 1∶0.25. The particle size of all formulations was around 250 nm, regardless the amount of antigen initially included, although slightly larger if compared to blank CSNC (**Table 1**). Additionally, it was observed that the association efficiency increased with the amount of HB added, reaching a value of around 80% of HB for the ratio CSNC:HB 1∶0.25. Despite this high association rate, the ξ potential of the nanocapsules remained highly positive (+40 mV), thus, indicating the prevalence of the cationic polysaccharide on the surface of the nanostructure. On the other hand, increments in the antigen concentration beyond 0.25 mg/mL led to unstable nanosystems.

**Figure 2:**
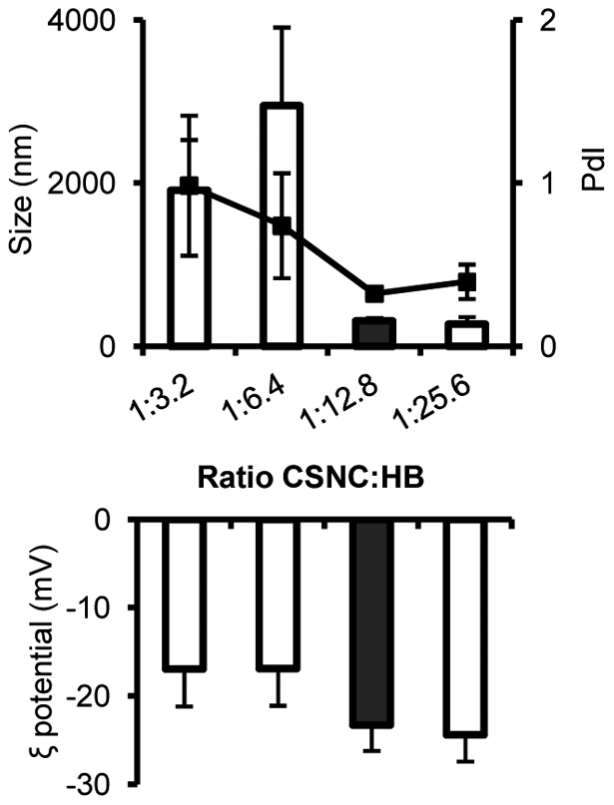
Physicochemical characterization of anionic HB-surface assembled CSNC prototypes. Particle size, polydispersion (PdI) and ξ potential evolution of CSNC:HB when CSNC suspension was sequentially diluted. The ratio CSNC:HB which exhibited the most adequate physicochemical properties (black column) was: particle size 310 nm ±34; polydispersity index 0.33±0.04; ξ potential −23 mV ±3. Mean ± SD.

In order to further explore the association of the antigen to CSNC, we chose an alternative incubation protocol. A suspension of blank CSNC was sequentially diluted and then incubated with constant antigen amount (25 μg). Using this method, we observed an inversion of the surface charge of the nanosystem for the ratio CSNC:HB, 1∶3.2. Although, as shown in **Figure 2**, this inversion was initially accompanied by a significant size increment (larger than 1μm), it was possible to preserve the nanometric size by reducing the concentration of CSNC. Indeed, for the CSNC:HB ratio of 1∶12.8, we obtained nanostructures of a size around 310 nm, a negative surface charge (−20 mV) and high association efficiency (83%).

Therefore, two different prototypes were obtained with similar particle size, but opposite surface characteristics in terms of composition and ζ potential: CSNC:HB with ratios of 1∶0.25 (CSNC+) and 1∶12.8 (CSNC−).

### Influence of surface charge in the adjuvant capacity of HB-surface-assembled CSNC prototypes

In order to assess the adjuvant capacity of CSNC+ and CSNC−, we administered the anionic and cationic prototypes by intramuscular (i.m.) injection to female BALB/c mice. Briefly, prime and boost doses of 10 µg of HB associated to CSNC, as well as to aluminium hydroxide (conventional vaccine: HB-alum) at the same dose, were given in a 4 week interval.

**Figure 3:**
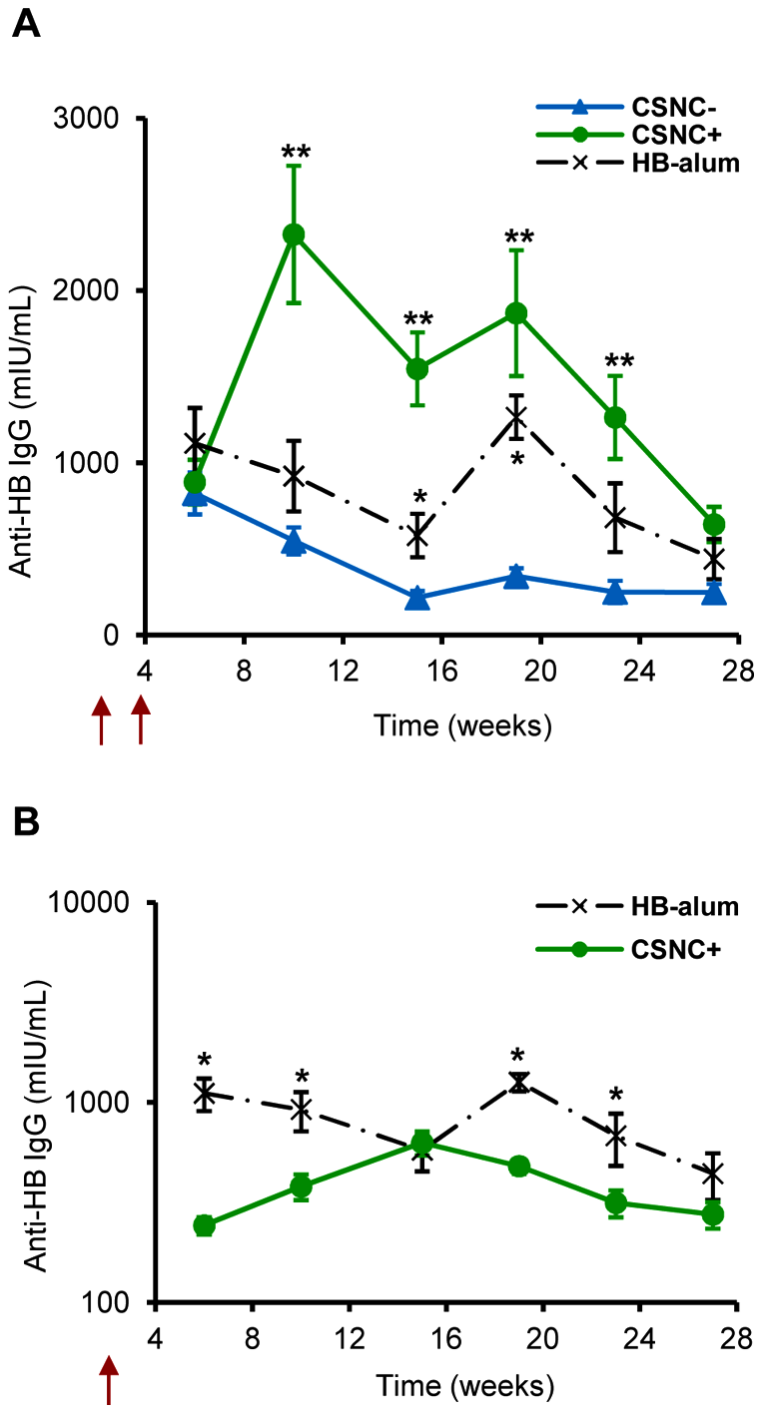
Evaluation of the efficacy of HB-surface assembled CSNC prototypes. Immune response induced by the nanovaccine prototypes (CSNC− and CSNC+) and the positive control (HB-alum) administered intramuscularly following different vaccination protocols. (**A**) IgG anti-HB levels generated after prime-boost immunization (arrows, weeks 0–4) with both prototypes: CSNC− (blue –▴–); CSNC+ (green –•–) and HB-alum (black –·X·–) at the same dose (10 µg). Results are presented as mean ± SEM.*Alum or CSNC+ vs. CSNC− (p<0.05). ** CSNC+ vs. Alum and CSNC- (p<0.05). (**B**) IgG anti-HB (mIU/ml) levels elicited after a single dose (arrow) of CSNC+ (10 µg; green –•–) compared to alum-HB administered twice (10 µg, weeks 0 and 4) (black –·X·–). Results are presented as mean ± SEM.*(p<0.05).

As observed in **Figure 3A**, CSNC− was not able to induce a significant response against HB as compared to the vaccine containing alum, although anti-HB IgG levels remained over seroprotective levels (>10 mUI/mL for humans [23]) during the 27-week study. In contrast, the cationic formulation (CSNC+) was able to generate a more potent antibody response than that elicited by the HB-alum at the same dose (p<0.05), thus, proving the immune potentiating effect of CSNC+. These results represent the proof-of-concept of the adjuvant capacity of HB-surface assembled CSNC+ prototype.

### Induction of immune protection after a single injection of cationic HB-surface- assembled CSNC prototype

Taking into account the adjuvant effect observed for the CSNC+, we decided to evaluate the immune response elicited by this prototype after a single administration (10 µg) and compare it to two doses of HB-alum (10 µg at 0 and 4 weeks). As noticed in **Figure 3B**, although slightly lower, the antibody levels induced by only one shot of CSNC+ were similar to those elicited upon vaccination with two doses of 10 μg of HB-alum. In fact, no significant differences were observed between the vaccination regimens, except for two time-points (at 5 and 19 weeks post-immunization) (p<0.05). Additionally, a single dose of CSNC+ induced protective anti-HB IgG levels (>10 mU/ml in humans), which remained around 200 mIU/mL until the end of the study (week 27). IgG levels higher than 100 mIU/mL are considered to provide proper immune protection against HB, suggesting that no additional boosters are further required [23].

### Effect of cationic HB-surface assembled CSNC on the modulation of the elicited immune response

The effect of CSNC+ on the type of T helper (Th1 or Th2) responses preferentially involved was evaluated by measuring the serum anti-HB IgG subtypes (IgG1 and IgG2a) and calculating the ratio between both. While higher levels of antigen specific IgG1 in mice are associated to a predominant humoral-Th2 type response, the presence of IgG2a is mostly related to a cellular-Th1 type.

**Figure 4:**
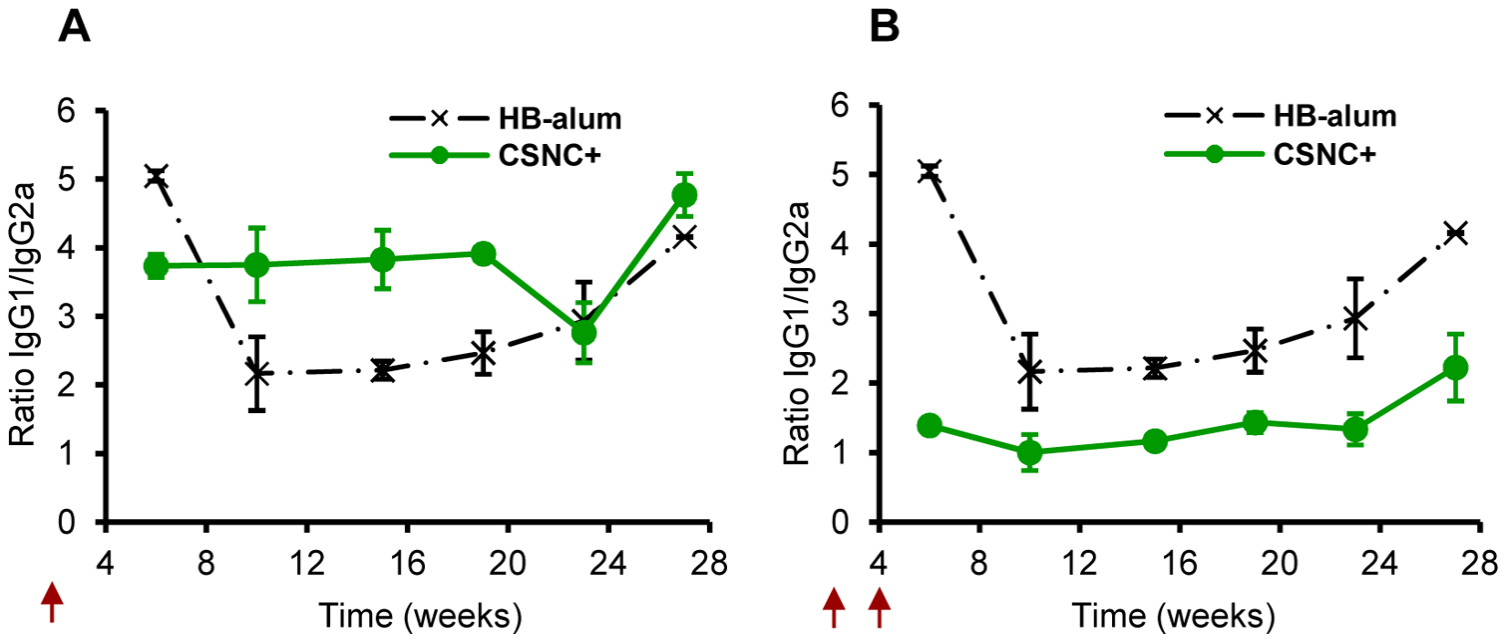
Modulation of the immune response by CSNC+. Ratio of IgG1/IgG2a anti-HB for prototype CSNC+ (10 μg; green –•–) after single (**A**) or double immunization separated 4 weeks (**B**) (indicated in arrows) compared to HB-alum (10 μg) administered in two doses (0, 4 weeks) (black –·X·–). Results are presented as mean ± SD.

IgG1/IgG2a ratios represented in **Figure 4** indicate that HB-alum vaccine induced a predominant humoral Th2-type response (higher levels of IgG1 with IgG1/IgG2a ratios ranging from 2 to 5), as expected [24]. Higher production of IgG1 than IgG2a was also found after a single immunization with CSNC+. However, a second administration of this nanovaccine lessened the IgG1/IgG2a ratio, suggesting a cellular immune response (Th1 type) was also induced. These results show the impact of the nanovaccine booster upon the modulation of the immune response by CSNC+.

### Stability of the aqueous suspension of cationic HB-surface-assembled CSNC prototype

The stability of the prototype CSNC+ as aqueous suspension was assessed during its storage at 4°C. The particle size, polydispersity index and HB association were analyzed each week during one month. The nanosystem maintained its original particle size and homogeneous distribution during at least one month of storage under refrigerated conditions (**Figure S2**). Similarly, the antigen remained associated to CSNC during this period.

### Freeze-drying of cationic HB-surface-assembled CSNC prototype

**Figure 5:**
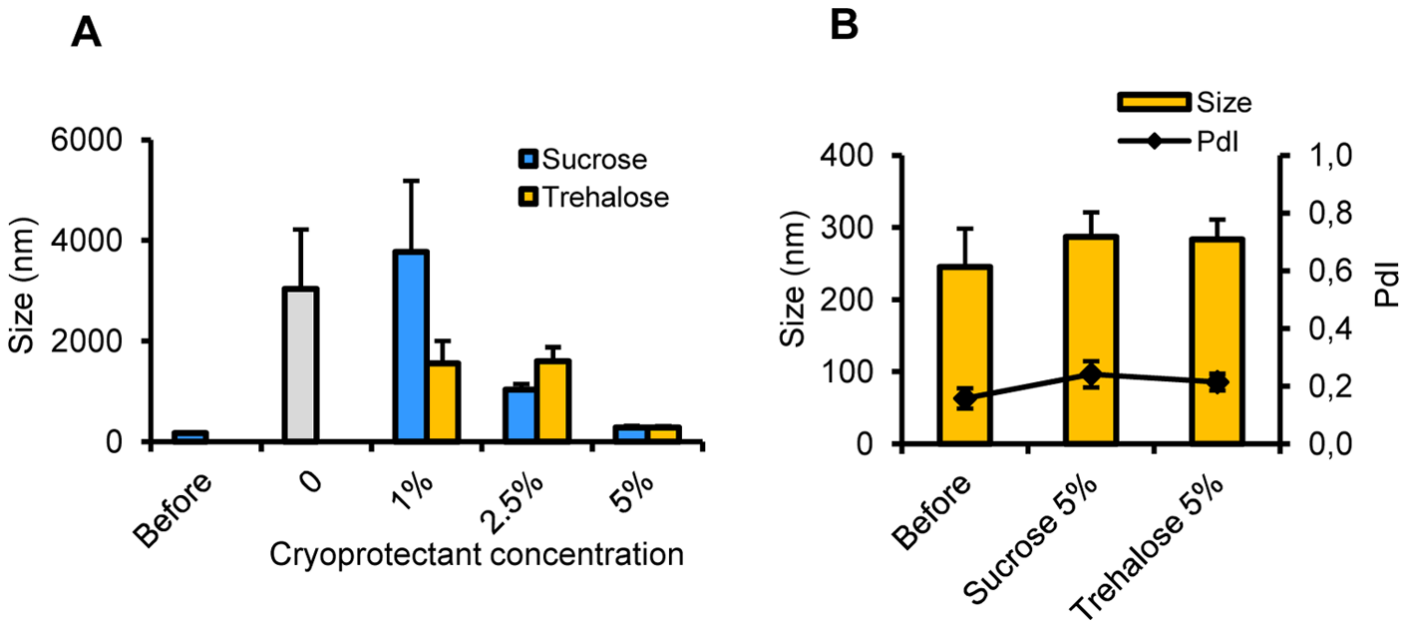
Physicochemical characterization of reconstituted CSNC+ freeze dried under different conditions. Particle size recovery after freeze-drying and reconstitution of CSNC+ using different cryoprotectants. (**A**) Particle size after reconstitution of freeze-dried formulation using sucrose (blue columns) and trehalose (yellow columns) as cryoprotectants at different concentrations. (**B**) Detailed physicochemical characterization of dried formulations in the presence of sucrose or trehalose (both at 5%) compared to the original characteristics of the suspension (before). Size is shown in yellow columns and polydispersity index (PdI) in black line. Results are presented as mean ± SD.

The aqueous suspension of CSNC+ was transformed into a dried powder using standard freeze-drying techniques. Two different cryoprotectants, sucrose and trehalose, at increasing concentrations were added to the CSNC+ suspension to achieve a final concentration of 1, 2.5 or 5%. After freeze-drying, the resulting dried cakes had an overall good appearance without signs of collapse. Samples were rehydrated and redispersed without appreciable macroscopic aggregates. Particle size of the reconstituted suspension was then analyzed. On those samples without cryoprotectant or with low sugar concentration (1 and 2.5%), the particle size was found to be much larger compared to their sizes before freeze-drying (**Figure 5**). At 5% sucrose or trehalose, the original particle size of the fresh suspension was properly recovered.

Trehalose is usually preferred for cryoprotection of biomacromolecules because of its lower hygroscopicity, capacity of formation of more flexible hydrogen bonds and very low reactivity [25]. Because the HB was associated to the CSNC surface and therefore more exposed to freezing stress, this disaccharide was selected as the most adequate cryoprotectant to produce a freeze-dried product from this nanosystem suspension when included in a concentration of 5%.

### Immunization with freeze-dried cationic HB-surface-assembled CSNC

To assess the preservation of the adjuvant properties of CSNC and immunogenicity of associated antigen after freeze-drying, dried CSNC+ with trehalose 5% was properly reconstituted and administered to BALB/c mice. Animals were intramuscularly vaccinated at 0 and 4 weeks with either the reconstituted CSNC+ prototype or freshly prepared HB-alum (as positive control), both containing 10 µg of HB. Dried CSNC+ was able to induce IgG titers similar to those achieved by standard alum-based vaccine (**Figure S3**). This result indicates that the freeze-dried product preserved a marked adjuvant capacity after the freeze-drying process and during transportation and storage without cold chain restrictions.

## Discussion

Nanocapsules are colloidal structures showing a characteristic core-corona architecture, consisting of an oily liquid core surrounded by a polymeric shell. Until now, this type of structure has been used for the encapsulation of drugs with the idea of improving their solubility, as well as their transporting through mucosal barriers [26]. Herein, the technology of chitosan nanocapsules (CSNC) was adapted to explore its potential for antigen delivery. Particularly, we studied the possibility to entrap and present the antigen molecules onto the surface of the nanocarrier and the influence of the physicochemical properties of the resulting nanovaccine on the generation of specific immune responses. Given the multifunctional character of these nanocarriers, the results of this work are expected to lead the way for their rational optimization based on the association of different immunostimulants and antigens.

Two different HB-surface-assembled CSNC prototypes were developed by modifying the proportions between CSNC and the antigen. CSNC:HB with ratios of 1∶0.25 (CSNC+) and 1∶12.8 (CSNC−) were obtained with similar particle size, but opposite surface characteristics in terms of composition and ζ potential (**Table 1** and **Figure 2**). The cationic surface charge of CSNC+ indicated the prevalence of CS on the surface of the nanostructure despite the high antigen association rate. In contrast, the negative charge of CSNC− indicated the exposure of the antigen molecules entrapped in the CS shell towards the external medium. This surface antigen display approach has been proposed as a way to mimic the natural structure of pathogens [27]. In fact, the exposure of repetitive copies of the antigenic epitope on the surface of nanoparticles has been demonstrated to enhance the immune response against weakly immunogenic antigens [28]. In addition, the surface charge of the nanocarriers has been shown to affect their adjuvant properties, and in particular, the cationic surface charge has been determined to enhance the immune responses [29]. Therefore, the influence of the antigen exposure and the surface properties (charge and composition) of the nanocarriers on the immune response was then investigated.

The immune response observed for CSNC+, CSNC− and the control HB-alum administered following a boost-dose immunization schedule evidenced clear differences between both CSNC prototypes. Namely, CSNC−, which contained a high proportion of antigen molecules on the surface of the nanostructure, was not efficient at improving the immune response elicited by HB-alum, suggesting that the presentation of repetitive copies of HB molecules onto a particulate carrier is not enough to trigger a potent immune response and other characteristics of the nanostructure are more crucial. In contrast, the prototype exhibiting an excess of CS on their surface (CSNC+), was able to stimulate the generation of a pronounced IgG response against HB, which was even higher than for the HB-alum (**Figure 3A**). This positive effect of the CS coating being exposed on the surface is in agreement with the promising results recently reported by our group for nanogelled CS encapsulating HB [14]. The intense humoral immune response achieved with both CS-based nanocarriers provides additional evidence of the immunostimulatory properties of nanostructured CS [14], [18]–[21].

The adjuvant properties of CS itself have been previously studied *in vivo* with other model antigens, such as β-galactosidase [30]. The adjuvant effect of CS was mainly attributed to its capacity to form a depot at the injection site *in vivo* [30] and also to attract immunocompetent cells to the area [31]. On the other hand, some studies have raised the issue of a possible direct immunostimulation mechanism of CS through the Toll-like receptor 4 (TLR4) [32], [33]. Unfortunately the results published are inconclusive due to the different nature and purity of the CS used in these studies. Irrespective of the potential inherent immunostimulatory behavior of CS, it is known that the association of antigens to particulate carriers facilitates their uptake by APCs and subsequent activation [4], [5]. In particular, nanostructures composed of different cationic biomaterials, such as cationized gelatin [34], poly-L-lysine or protamine [35], have been shown to positively promote recognition and uptake by APCs. Likewise, CS-based nanoparticles increased antigen internalization due to their strong association to the outer membrane of the dendritic cells [36]. Therefore, the association of HB to CSNC+, characterized by a cationic surface charge and predominant presence of CS on their surface, could have importantly contributed to its adequate presentation to the immune system and moreover, to an enhanced activation of APCs and further development of a strong adaptive immune response.

One of the main goals in vaccination has been to reduce the number of injections but achieving an efficient immune protection. Indeed, a vaccine able to generate long-term immune responses after a single immunization offers many advantages, such as reducing the risk of patients' under-protection due to a deficient compliance to the immunization schedule, minimizing waste disposal, and decreasing the costs related to vaccination [5]. The sustained effect observed for CSNC+ after a single administration (**Figure 3B**) could be attributed to a prolonged antigen delivery to peripheral APCs possibly due to an accumulation of the nanostructures at the injection site (depot effect) and subsequent drainage to the lymph nodes. Additionally, the strong interaction between HB and the polysaccharidic shell of CSNC may have also contributed to this effect, enhancing the retention of the associated antigen and enabling its cellular uptake for long periods of time [37]. These results provide the first evidence of the ability of CSNC to elicit high specific, long-lasting and protective IgG levels against HB after a single-dose vaccination regimen.

To study the effect of CSNC+ prototype on modulating the elicited immune response, IgG1 and IgG2a levels were analyzed in sera collected from vaccinated mice. The ratio IgG1/IgG2a is used as indicator of the predominant immune response and cells involved (predominant Th1 or Th2 immune response). The type of immune response elicited by CSNC+ was strongly dependent on the immunization regimen. A single dose with CSNC+ resulted in a predominant Th2-mediated response (humoral), whereas both Th1- and Th2-type responses were induced after booster immunization (**Figure 4**). This type of response was also reported for CS aqueous solutions mixed with model antigen (β-galactosidase) [30] and for ovalbumin-loaded CS nanoparticles administered subcutaneously [38]. Overall, these findings suggest the potential of CS and CS nanocarriers to promote a mixed Th1/Th2 immune response.

A critical feature of an antigen delivery carrier is its ability to preserve the stability of the associated antigen during storage. Initially in this study, we observed CSNC+ maintained its physicochemical properties for one month during storage under refrigerated conditions (**Figure S2**). In a second instance, we explored the freeze-drying method as a way to improve long-term stability of colloidal formulations and avoid cold chain restrictions. In a dry environment, nanostructures and the associated bioactive molecule can be protected from degradation, while their original physicochemical properties can be recovered upon rehydration [39].

The results evidenced the feasibility to transform the aqueous suspension of CSNC+ into a dry powder that can be rehydrated, while maintaining the original physicochemical properties (**Figure 5**). Furthermore, upon reconstitution, the dried formulation maintained the adjuvant properties following *in vivo* administration (**Figure S3**). As the dry form may be a suitable presentation for long-term storage, the preservation of the immune behavior of the nanovaccine upon freeze-drying is critical [40]. This is indeed an advantage over common aluminum salts that cannot be frozen and consequently freeze-dried. The particulate structure of alum can be destroyed after freezing, leading to a loss of potency of the vaccine [41], which is an important drawback leading to the necessity of strictly maintaining the cold chain during transportation and storage, a particularly difficult requirement for developing countries with deficient logistic infrastructures [5], [42].

The results of this work represent the first proof-of-principle of the potential of CSNC as antigen delivery adjuvants, in particular for a vaccine using the HB antigen as model antigen. Furthermore, these results provide preliminary evidence of the value of this technology as a single-dose immunization strategy against the disease. With this first study, we were able to establish the optimal organization of the antigen molecules on the nanocarrier's surface to enhance the specific immune response using the CSNC platform. However, from the multifunctional structure of the CSNC it could also be deduced that further improvements could be achieved by incorporating additional immunostimulants in the CSNC's core. In addition, this new technology may also represent a way to preserve the stability of the antigen in a freeze-dried form. Finally, from the conceptual point of view, the results of this work further assess the value of positively charged nanocarriers as a way to facilitate antigen presentation to the immune cells.

## Supporting Information

Figure S1 **Illustration of the different preparation protocols to obtain both HB-surface-assembled CSNC prototypes.** (**A**) CSNC+ (ratio CSNC:HB 1∶0.25) and (**B**) CSNC− (ratio CSNC:HB 1∶12.8).
(TIF)

Figure S2 **Stability of CSNC+.** Both particle size (blue columns) and percentage of associated HB to CSNC (dark blue line) are shown at different time points (0–4 weeks) during storage at 4°C. Results are presented as mean ± SD.
(TIF)

Figure S3 **Efficacy of freeze-dried CSNC+ upon storage and reconstitution.** Humoral immune response (IgG titers) after two i.m. administrations of reconstituted freeze-dried CSNC+ (green –•–) and compared to HB-alum (black –·X·–) at the same dose (10 µg, weeks 0 and 4). Results are presented as mean ± SEM.
(TIF)

Protocol S1 **Solvent displacement technique protocol for the preparation of blank CSNC.**
(DOCX)
