# Supplementary figures and images for: A Polymer/Oil Based Nanovaccine as a Single-Dose Immunization Approach
Source: PLoS One. 2013 Apr 22;8(4):e62500. doi: 10.1371/journal.pone.0062500 (PMC3632546; doi:10.1371/journal.pone.0062500)

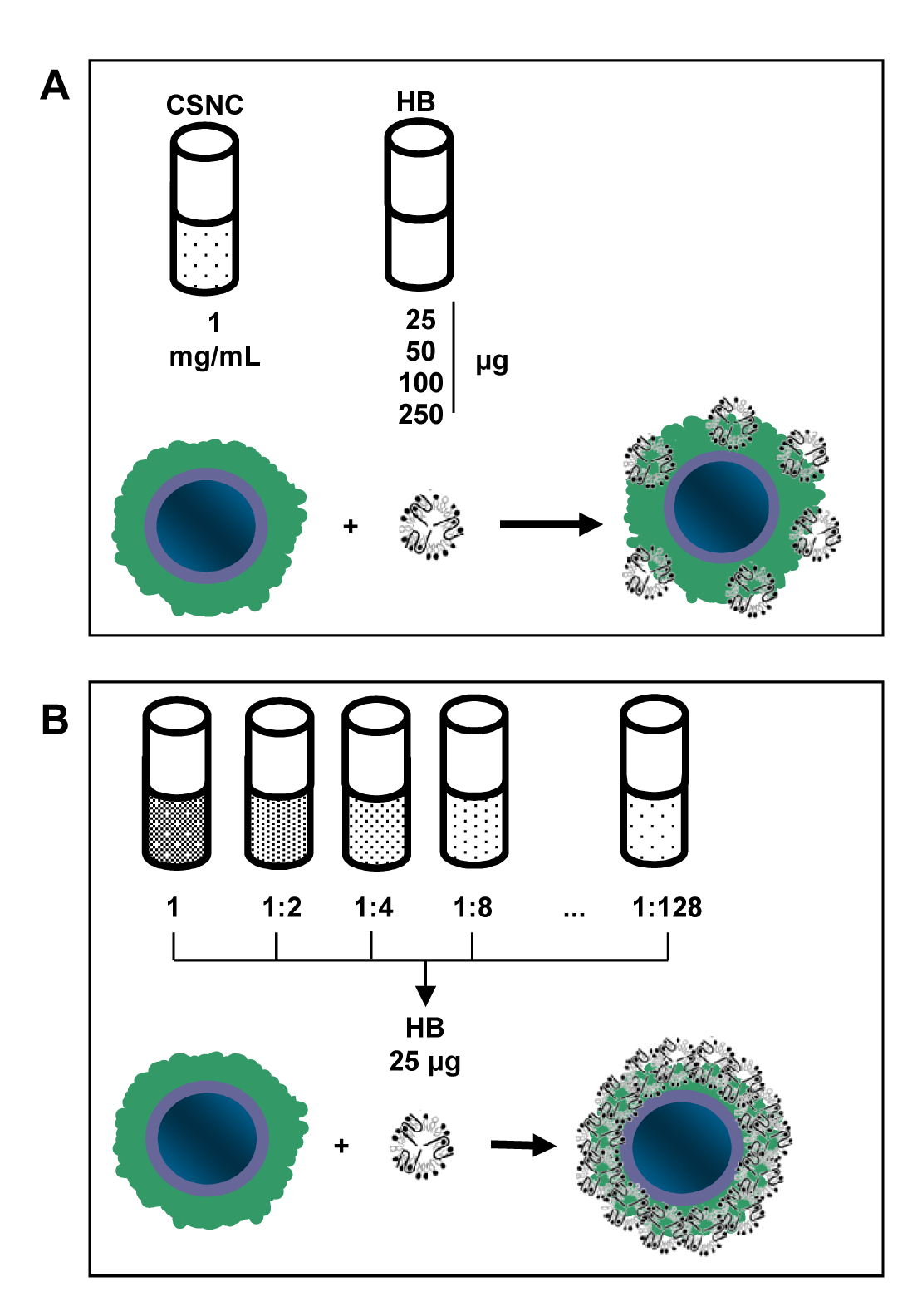

Supplement: Figure S1 — Illustration of the different preparation protocols to obtain both HB-surface-assembled CSNC prototypes. (A) CSNC+ (ratio CSNC:HB 1∶0.25) and (B) CSNC− (ratio CSNC:HB 1∶12.8). (TIF) [file pone.0062500.s001.tif]

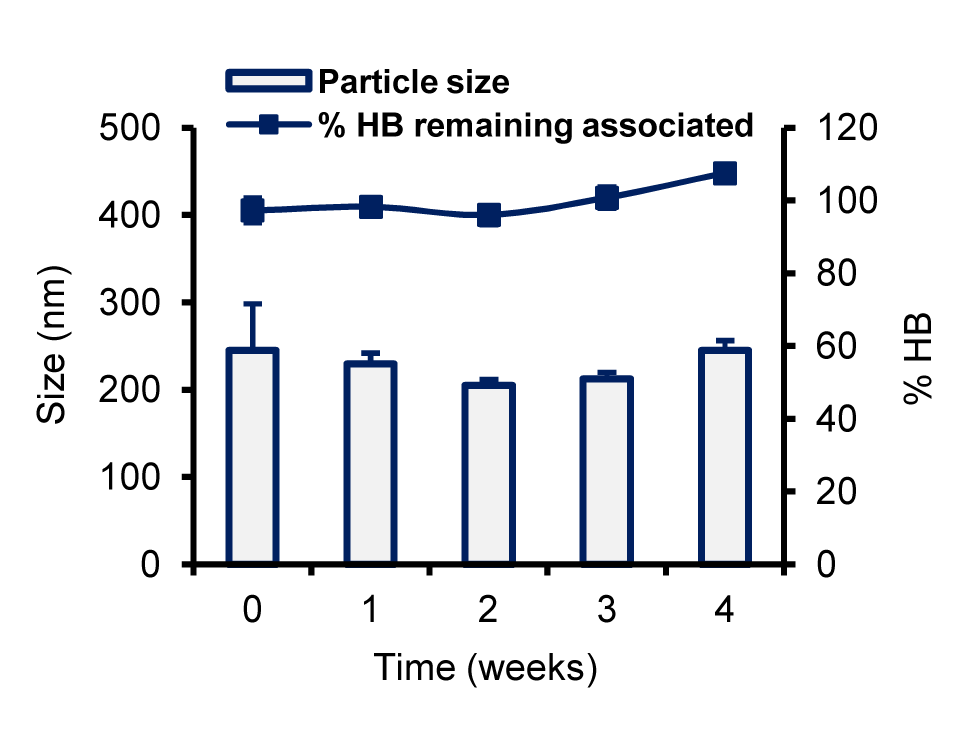

Supplement: Figure S2 — Stability of CSNC+. Both particle size (blue columns) and percentage of associated HB to CSNC (dark blue line) are shown at different time points (0–4 weeks) during storage at 4°C. Results are presented as mean ± SD. (TIF) [file pone.0062500.s002.tif]

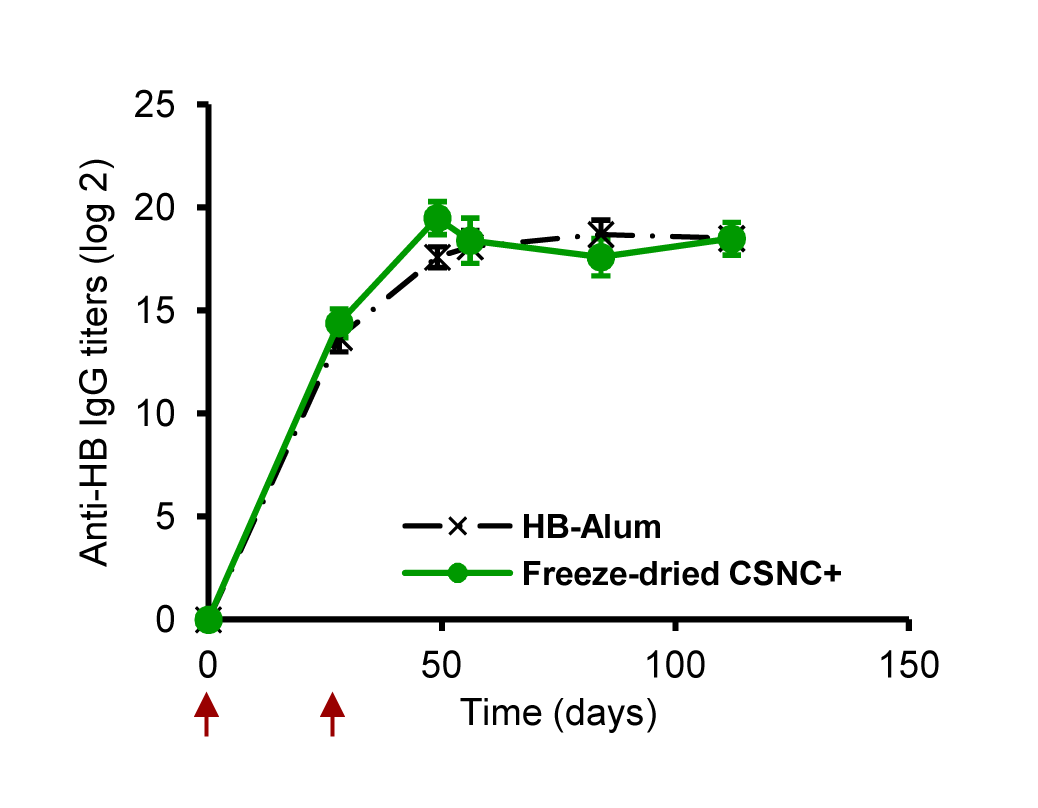

Supplement: Figure S3 — Efficacy of freeze-dried CSNC+ upon storage and reconstitution. Humoral immune response (IgG titers) after two i.m. administrations of reconstituted freeze-dried CSNC+ (green –•–) and compared to HB-alum (black –·X·–) at the same dose (10 µg, weeks 0 and 4). Results are presented as mean ± SEM. (TIF) [file pone.0062500.s003.tif]
