# Supplementary material for: A Polymer/Oil Based Nanovaccine as a Single-Dose Immunization Approach
Source: PLoS One. 2013 Apr 22;8(4):e62500. doi: 10.1371/journal.pone.0062500 (PMC3632546; doi:10.1371/journal.pone.0062500)
Supplement: Protocol S1 — Solvent displacement technique protocol for the preparation of blank CSNC. (DOCX) [file pone.0062500.s004.docx]

**Protocol S1: Solvent displacement technique protocol for the preparation of blank CSNC**

Briefly, 40 mg and 0.25 mL of M812 were dissolved in a mixture of ethanol and acetone. This organic phase was poured under magnetic stirring upon an aqueous solution composed of CS 0.05% in water. CSNC were immediately formed, as noted by the milky appearance of the resulting solution. Then, the organic solvents were evaporated under vacuum. Finally, CSNC suspension was isolated by ultracentrifugation (Optima^TM^ L-90K Ultracentrifuge, Beckman Coulter; Fullerton, CA) at 42000xg for 1 hour at 15ºC and then resuspended in water to a final concentration of CS in the formulation of 1 mg/mL.
